# Various aspects of Kikuchi disease in three children: systemic or self-limited disease?

**DOI:** 10.1186/1546-0096-9-S1-P235

**Published:** 2011-09-14

**Authors:** L Goffin, S Huybrechts, C Heijmans, AS Bouteiller, C Thomée, V Segers, MF Dehou, A Ferster

**Affiliations:** 1 Department of Pediatry, Hôpital Universitaire Des Enfants Reine Fabiola, Brussels, Belgium; 2 Department of Pediatry, Centre Hospitalier de Luxembourg, Luxemburg; 3 Department of pathology, CHU Brugmann, Brussels, Belgium

## 

Kikuchi’s disease (KD), or histiocytic necrotizing lymphadenitis, is a rare benign and self-limited disease involving young adults, predominantly females. It is rarely described in children. It is characterized by localized lymphadenopathy, often associated with fever and systemic symptoms. The diagnosis is based on histological examination of lymph node biopsy. The disease usually resolves spontaneously over a period of several weeks to months. In some cases, KD reveals or evolves into a systemic lupus, reason why long term follow-up is recommended.

Three cases of pediatric KD are presented in Table 1.

**Table 1:** 

	Patient 1	Patient 2	Patient 3
**Age (years)**	5	17	9

**Underlying disease**	Non complicated sickle cell anemia	Severe sickle cell anemia	None

**Symptoms**	- Left cervical lymphadenopathy - Intermittent fever	- Intermittent fever - Maculo-papules - Fatigue - Painful cervical and inguinal adenopathies - Headaches - Polyarthritis	- Prolonged fever - Intermittent tonsillitis - Bilateral painful cervical lymph nodes

**Biological features**	- Mild inflammation - Elevated LDH	- Marked inflammation - Pancytopenia - Auto-immune anemia - Auto-immune hepatitis - No ANA	- Marked inflammation - Leucopenia

**Biopsy**	Cervical lymph node excisional biopsy	Cervical lymph node excisional biopsy	Tonsillectomy

**Treatment**	None	- Steroids for 7 months - Azathioprine - Methotrexate	None

**Outcome**	Spontaneous resolution after 4 months	Progressive regression of clinical and biological signs with resolution of auto-immunity	Spontaneous resolution 2 months after tonsillectomy

In conclusion, KD is rarely observed in children, has various presentations but usually favorable outcome. This small cohort of pediatric patients illustrates this diversity: one of them presented with marked systemic symptoms, suggesting SLE but resolving after prolonged corticotherapy, while the others had a more benign course with spontaneous resolution after excision. Association with sickle cell disease has not been described and, to our best knowledge, this is the first case diagnosed on tonsil examination.

